# Development of a patient decision aid for patients with breast cancer who consider immediate breast reconstruction after mastectomy

**DOI:** 10.1111/hex.13368

**Published:** 2021-10-28

**Authors:** Jacqueline A. ter Stege, Daniela B. Raphael, Hester S. A. Oldenburg, Martine A. van Huizum, Frederieke H. van Duijnhoven, Daniela E. E. Hahn, Regina The, Klemens Karssen, Eveline M. L. Corten, Irene S. Krabbe‐Timmerman, Menno Huikeshoven, Quinten (P. Q.) Ruhé, Nikola (A. N.) Kimmings, Wies Maarse, Kerry A. Sherman, Arjen J. Witkamp, Leonie A. E. Woerdeman, Eveline M. A. Bleiker

**Affiliations:** ^1^ Psychosocial Research and Epidemiology Netherlands Cancer Institute, Antoni van Leeuwenhoek Amsterdam The Netherlands; ^2^ Radiation Oncology (Maastro) GROW School for Oncology and Developmental Biology, Maastricht University Medical Center Maastricht The Netherlands; ^3^ Radiotherapy Netherlands Cancer Institute, Antoni van Leeuwenhoek Amsterdam The Netherlands; ^4^ Surgical Oncology Netherlands Cancer Institute, Antoni van Leeuwenhoek Amsterdam The Netherlands; ^5^ Plastic and Reconstructive Surgery Netherlands Cancer Institute, Antoni van Leeuwenhoek Amsterdam The Netherlands; ^6^ Psychosocial Counseling Netherlands Cancer Institute, Antoni van Leeuwenhoek Amsterdam The Netherlands; ^7^ ZorgKeuzeLab Delft The Netherlands; ^8^ Plastic and Reconstructive Surgery Erasmus Medical Center Rotterdam The Netherlands; ^9^ Plastic and Reconstructive Surgery Franciscus Gasthuis & Vlietland Rotterdam The Netherlands; ^10^ Plastic and Reconstructive Surgery Medical Center Leeuwarden Leeuwarden The Netherlands; ^11^ Plastic and Reconstructive Surgery Spaarne Gasthuis Haarlem The Netherlands; ^12^ Plastic and Reconstructive Surgery Meander Medical Center Amersfoort The Netherlands; ^13^ Surgery Slotervaart Medical Center Amsterdam The Netherlands; ^14^ Plastic and Reconstructive Surgery University Medical Center Utrecht Utrecht The Netherlands; ^15^ Department of Psychology, Centre for Emotional Health Macquarie University Sydney Australia; ^16^ Surgery University Medical Center Utrecht, Utrecht The Netherlands; ^17^ Clinical Genetics Leiden University Medical Center Leiden The Netherlands

**Keywords:** breast cancer, immediate breast reconstruction, information needs, patient decision aid

## Abstract

**Purpose:**

The aim of this study was to develop a patient decision aid (pDA) that could support patients with breast cancer (BC) in making an informed decision about breast reconstruction (BR) after mastectomy.

**Methods:**

The development included four stages: (i) Establishment of a multidisciplinary team; (ii) Needs assessment consisting of semi‐structured interviews in patients and a survey among healthcare professionals (HCPs); (iii) Creation of content, design and technical system; and (iv) Acceptability and usability testing using a think‐aloud approach in patients and interviews among HCPs and representatives of the Dutch Breast Cancer Patient Organization.

**Results:**

From the needs assessment, three themes were identified: Challenging period to make a decision, Diverse motivations for a personal decision and Information needed to make a decision about BR. HCPs valued the development of a pDA, especially to prepare patients for consultation. The pDA that was developed contained three parts: first, a consultation sheet for oncological breast surgeons to introduce the choice; second, an online tool including an overview of reconstructive options, the pros and cons of each option, information on the consequences of each option for daily life, exercises to clarify personal values and patient stories; and third, a summary sheet with patients’ values, preferences and questions to help inform and guide the discussion between the patient and her plastic surgeon. The pDA was perceived to be informative, helpful and easy to use by patients and HCPs.

**Conclusion:**

Consistent with information needs, a pDA was developed to support patients with BC who consider immediate BR in making an informed decision together with their plastic surgeon.

**Patient or Public Contribution:**

Patients participated in the needs assessment and in acceptability and usability testing.

## 1 INTRODUCTION

Patients undergoing mastectomy as a treatment for breast cancer (BC) or to reduce their increased risk of BC often have a choice of whether or not to undergo breast reconstruction (BR). Undergoing BR after mastectomy can be beneficial for patients’ quality of life and psychosocial functioning.^1^, ^2^, ^3^, ^4^, ^5^ However, there are also disadvantages of having BR, such as an increased risk for complications.^6^, ^7^ Most patients who consider BR also have to make choices regarding the timing (i.e., immediate or delayed) and the type (i.e., implant‐based or autologous) of surgery.

The decision for BR largely depends on patients’ values and preferences.^8^, ^9^ For preference‐sensitive decisions such as this, shared decision‐making is increasingly advocated as the preferred approach.^10^, ^11^ Shared decision‐making is a patient‐centred approach in which physicians and patients collaborate and share information about the best available evidence and patient preferences and values to reach a health decision.^10^, ^12^, ^13^ In this approach, physicians are considered experts about the medical evidence and patients are considered experts about what matters most to them.^14^

Previous studies have suggested that there remains an unmet need for support in the context of decision‐making about BR after mastectomy since both knowledge and decisional preparedness are low among patients deciding about BR.^15^, ^16^, ^17^ Moreover, another study found that less than half (43%) of the participants made a high‐quality decision regarding BR, defined as having knowledge of important BR facts and undergoing treatment in accordance with one's personal preferences.^18^ Furthermore, previous studies found that a substantial number of women (37% up to 47%) experienced some level of decisional regret after undergoing BR.^19^, ^20^, ^21^ With a median time period between diagnosis and surgery of 5 weeks, patients often have limited time to decide about immediate BR.^22^ Previous studies have highlighted the importance of high‐quality, realistic preoperative information and decisional support to enable patients to make a long‐term satisfying decision about BR.^19^, ^20^, ^23^, ^24^, ^25^, ^26^, ^27^, ^28^

Patient decision aids (pDAs) may be beneficial for patients who are facing the decision regarding BR. PDAs are tools that, as adjuncts to counselling, aim to support shared decision‐making. PDAs explicitly state the decision, consist of evidence‐based information about the options and their pros and cons and clarify patients’ personal values.^29^ Across a variety of health‐related decisions, pDAs have been found to reduce decisional conflict, increase knowledge and increase insight into personal values related to the decision.^30^, ^31^

Worldwide, a limited number of pDAs are available for patients considering BR.^32^, ^33^ Whilst studies showed promising results regarding their effectiveness,^32^, ^33^ no evidence‐based pDA is available for patients considering BR in the Netherlands.

Therefore, the aim of this study was to develop an online pDA that could support patients in making an informed decision about BR after mastectomy together with their plastic surgeon. As part of the development of this pDA, we aimed to assess the information needs of both patients and healthcare professionals (HCPs) and to test the acceptability and usability of the pDA.

## 2 METHODS

**Figure 1:**
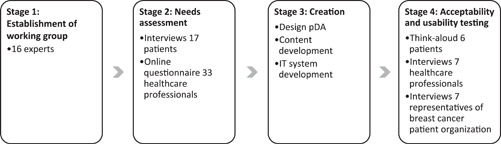
Overview of the four stages of pDA development and participants. pDA, patient decision aid

The development was guided by International Patients Decision Aids Standards (IPDAS) criteria for developing a high‐quality pDA.^34^ The development was performed in partnership with ZorgKeuzeLab, a Dutch company specialized in the development and implementation of pDAs. The development consisted of four stages, briefly described in the protocol of the trial to evaluate the pDA^35^ and described in more detail below. For a schematic overview of the four stages and the participants, see Figure 1. The development of the pDA started in May 2016 and was completed in March 2017.

### 2.1 Stage 1: Establishment of a working group

We assembled a national working group consisting of 16 experts including plastic surgeons, oncological breast surgeons, psychologists, researchers, industrial designers and an expert in the development and implementation of pDAs. In four meetings (one meeting in each development stage), the working group reached consensus on the aim and scope of the pDA, discussed the content of the pDA and agreed on the final version of the pDA.

### 2.2 Stage 2: Needs assessment

We performed a needs assessment among patients and HCPs to assess information and decision support needs regarding BR. The Medical Research Ethics Committee of the Dutch Cancer Institute examined the study protocol and concluded that the obligation to fulfil the specific requirements of the Dutch law for Medical Research Involving Human Subjects be waived (reference: METC16.0840). All patients provided informed consent.

#### 2.2.1 Patients

Semi‐structured interviews were conducted with women who previously faced the decision regarding whether to undergo BR after mastectomy. Participants were recruited through purposive sampling to reach a sample diverse in age, educational level, indication for mastectomy (i.e., BC or prophylaxis), the decision to undergo BR and treating hospital. Members of the working group identified eligible participants among their patients, and subsequently asked these patients for approval to be contacted for the study. Upon approval, patients received more detailed study information by phone and an information letter and informed consent form by email. Interviews took place face to face at the Netherlands Cancer Institute, at ZorgKeuzeLab or, if preferred by the patient, via telephone. A psychologist/researcher (J. A. t. S.) conducted all the interviews, sometimes accompanied by a member of ZorgKeuzeLab (R. T. or K. K.). Interviews lasted approximately 60 min (see Supporting Information Appendix S1 for the complete interview script). Interviews were audio‐recorded, transcribed verbatim and coded by two independent researchers (J. A. t. S. and D. R.) using thematic analysis.^36^ Consensus about the coding scheme was reached in two consecutive meetings. Data were stored and coded in NVivo 10 (QSR International Pty Ltd.).

#### 2.2.2 Healthcare professionals

Forty HCPs who were involved in the BR decision‐making process were invited to complete a brief (15 min) study‐specific online questionnaire. HCPs included members of the working group and their colleagues from both within and beyond their hospital. In the questionnaire, HCPs were asked about their experiences and satisfaction with information about BR, their experiences and attitudes towards shared decision‐making and pDAs and their preferences for the content and implementation of the pDA to be developed. We performed descriptive analyses in IBM SPSS Statistics for Windows, Version 22 (IBM Corp.).

### 2.3 Stage 3: Creation

The central question for designing the pDA was as follows: ‘How can the pDA improve the conversation between a patient and a plastic surgeon about the decision for BR?’ (Including, what should a patient know about BR before consultation with a plastic surgeon? What should a plastic surgeon know about a patient regarding the diagnosis, values, preferences, circumstances and any other aspect relevant for decision‐making about BR before making a decision together?). Discussion about these questions within the working group guided the design of the pDA. The content was written by a team of physicians based on the guidelines for BR,^8^ the Stage 2 needs assessment results and discussion within the working group. Content was reviewed by working group members. A text writer edited texts at the B1 language level. Texts written in the B1 language level are considered as ‘fairly easy to read’ and are characterized by the use of common words and short, simple and active sentences.^37^ It is the recommended language level for public communication by the Dutch government as the vast majority of the population is able to understand it.^38^ The online infrastructure was built as an extension of an existing platform of pDAs (https://zorgkeuzelab.nl/keuzehulpen).

### 2.4 Stage 4: Acceptability and usability testing

The acceptability and usability of the developed pDA was assessed in patients who previously considered undergoing BR after mastectomy, HCPs involved in decision‐making about BR and representatives of the Dutch Breast Cancer Patient Organization (Borstkankervereniging Nederland). In *patients*, we used a ‘think‐aloud approach’, in which they were invited to literally think aloud whilst using the pDA.^39^ This is a common method for testing ICT tools including pDAs,^40^, ^41^, ^42^ and enables to get an impression of how patients perceive of and use the pDA. Each session finished with a short interview to evaluate the pDA (see Supporting Information Appendix S2 for the script). A total of eight patients who participated in the needs assessment and agreed to be contacted for acceptability and usability testing were invited. This procedure was performed at either ZorgKeuzeLab, the Netherlands Cancer Institute or via Skype. *HCPs* and *Representatives of the Dutch Breast Cancer Patient Organization* received access to the tool and were interviewed via telephone about their experiences with the pDA (see Supporting Information Appendix S2 for the script). HCPs who participated in the needs assessment and agreed to be contacted for acceptability and usability testing were invited. Representatives of the Dutch Breast Cancer Patient Organization, who had either previously considered BR after mastectomy or had expertise in pDAs, were recruited via the organization's project leader on shared decision‐making and via a call in a private Facebook group of the organization. The sessions and interviews (between 30 and 60 min each) took place between January and March 2017, and were performed by J. A. t. S. in the presence of a member of ZorgKeuzeLab (R. T. or K. K.). Major issues that hindered intended use of the pDA were modified directly upon identification. Notes and observations were combined and labelled as either general comments about the pDA or related to a specific section of the pDA. Feedback was presented to the working group, combined with suggestions for change. The working group members collaboratively decided upon the desired adjustments to the pDA.

## 3 RESULTS

### 3.1 Needs assessment

Seventeen patients (85%) and 33 HCPs (83%) participated in the needs assessment. Background characteristics of both groups are provided in Table 1.

**Table 1:** Background characteristics of participants in needs assessment

	N (%)
**Patients (*N* = 17)**	
Age (years), *M* (SD), range	51.3 (12.3), 31–77
Educational level	
High (higher vocational/university)	10 (59%)
Intermediate (secondary school/intermediate vocational)	7 (41%)
Low (primary school/lower vocational)	0 (‐)
Married or in a relationship	12 (71%)
Indication for mastectomy	
Breast cancer	14 (82%)
Prophylaxis	3 (18%)
Time since mastectomy (months), *M* (SD), range	46 (48), 2–173
Time since (last) reconstructive surgery (months), *M* (SD), range	23 (29), 2–115
Breast reconstruction (yes)	14 (82%)
Timing of breast reconstruction	
Immediate	11 (79%)
Delayed	3 (21%)
Type of breast reconstruction^a^	
Implant	9 (64%)
Autologous	6 (43%)
Combination	1 (7%)
Hospital^b^	
(Breast) cancer‐specific hospital	9 (53%)
General hospital	3 (18%)
Academic medical center	5 (29%)
**Healthcare professionals (*N* = 33)**	
Sex (female)	23 (70%)
Age (years), *M* (SD)	45.6 (8.2)
Profession	
Oncological breast surgeon	6 (18%)
Plastic surgeon	19 (58%)
Nurse (specialist/practitioner)	2 (6%)
Psychologist	4 (12%)
Social worker	2 (6%)
Number of years working in profession, *M* (SD)	13.8 (8.7)
Average number of new patients with breast cancer treated per month	
>30 patients	2 (6%)
11–30 patients	10 (30%)
1–10 patients	16 (49%)
None	5 (15%)
Organization^c^	
(Breast) cancer‐specific hospital	8 (24%)
General hospital	14 (42%)
Academic medical center	10 (30%)
Private practice	1 (3%)
Experience with referring patients to a patient decision aid (yes)	7 (21%)

Abbreviations: *M*, mean; SD,  standard deviation.

^a^ Numbers add up to more than 14 (number of patients with breast reconstruction) due to differences in the types of breast reconstruction for the left and right breasts.

^b^ Patients were recruited from five hospitals.

^c^ Professionals were recruited from 21 organizations.

#### 3.1.1 Patients

Thematic analysis yielded three themes reflecting patients’ most important experiences with, and information needs regarding, their BR decision (see Table 2 for illustrating quotes).

**Table 2:** Quotes illustrating experiences and information needs of patients deciding about breast reconstruction (*N* = 17)

Challenging period to make a decision
*At that time, you are mainly trying to survive and getting through your chemotherapy etcetera, you are totally not thinking of aesthetics at that time*. (Participant^4^, immediate, implant‐based BR) *At the moment, that we were inside* [the consultation room], *I guess your head is at another place. Because, there was little time in between. Mid‐June I was diagnosed, and mid‐July I already had surgery. So, in that short period, it had to be explained what was going to happen. But at that time, you are on another planet, so it seems. I did not at all absorb all information*. (Participant^5^, immediate, implant‐based BR)
Diverse motivations for a personal decision
*I think it is a very personal decision. I would suggest, discuss it with others… but well, you can discuss it with other people, but you are you. You have to live with it. You need to be happy with it*. (Participant^6^, no BR) *To not be flat. And to avoid the confrontation of a completely flat amputated breast. I knew that it [reconstructed breast] would have little of a breast when waking up [from surgery], but still, that you are not completely flat, and that you are not wearing a t‐shirt and have nothing on one side. That was very nice for me. That was also the reason for having it* [immediate BR]. (Participant^9^, immediate, implant‐based BR) *I have been through this [breast cancer], and as soon as I have finished this, I want to be done with it. I don't want any hassle on my body anymore, and I just want to exercise and get on with my life*. (Participant^6^, no BR)
Information needed to make a decision about breast reconstruction
*Information by women who have had it* [breast reconstruction], *you know, that would matter a lot. I never realized, of course you don't, that a prosthesis is cold. I don't have warm breasts anymore, but cold*. (Participant^4^, immediate, implant‐based BR) *That you can't walk straight in the first three weeks, but that you will walk like an old lady behind the walker. That are things that I actually only heard of, and experienced, after surgery*. (Participant^16^, immediate, autologous BR) *They say that you are allowed to do everything after six weeks* [after surgery], *but at that time, you can't do everything yet. You are still very limited. I could not carry my kids into the bath, or in their crib*. (Participant^14^, immediate, implant‐based BR)

Abbreviation: BR, breast reconstruction.

##### 3.1.1.1 Challenging period to make a decision

Patients with BC experienced the trajectory as a rollercoaster in which they were overwhelmed by emotions after a sudden diagnosis of BC. They had difficulties processing the large amount of information that they received. Some patients felt sick due to neoadjuvant systemic therapy and did not feel like themselves at the time of making their decision. Other patients highlighted the short period of time between diagnosis and surgery in which they had to make a decision, and the importance of taking adequate time to make a decision. Although many patients perceived having the option of BR as something positive, their highest priority at that time was to be cured from cancer, and aesthetics were less important. In contrast, women who considered undergoing BR after prophylactic mastectomy were not suddenly confronted with a diagnosis, did not feel sick and felt that they had sufficient time to become informed about BR and to make a decision. They stressed the importance of planning surgery at the right moment in their lives and of taking time to optimally prepare for surgery.

##### 3.1.1.2 Diverse motivations for a personal decision

Patients emphasized the importance of identifying their personal values to make a decision about BR. Although most patients had an immediate preference for or against undergoing BR, some patients had difficulties in making a decision. Patients’ reasons for their BR decision were diverse (see Table 3 for an overview of the reasons). The reasons for undergoing immediate BR included the desire to improve body image and appearance, and the reasons against undergoing immediate BR included having no interest in undergoing BR and the desire for faster recovery and avoiding increased risk for complications. The reasons for deciding on undergoing implant‐based BR included having no option for autologous BR and the desire for a shorter duration of surgery and faster recovery, and the reasons for autologous BR included the desire for more natural outcomes and avoiding the use of foreign materials. Although it was important to feel supported by their partner and relatives in making their decision, most patients emphasized that the decision had been made by themselves.

**Table 3:** Patients' reasons (A) for immediate versus against immediate breast reconstruction (B) for implants‐based versus autologous breast reconstruction

A. Reasons for immediate vs. against immediate breast reconstruction				
Immediate breast reconstruction^a^	*N*		Against immediate breast reconstruction^b^	*N*
Body image	6		No interest	5
Appearance	4		Faster recovery and avoid increased risk for complications	3
Avoid external prosthesis	3		Avoid scars and harms to other body parts	3
Less confrontation with cancer	2		Avoid foreign materials (implants)	2
Fewer surgeries than delayed reconstruction	2		Avoid surgery to replace implants (implants)	1
More clothing possibilities	1		Immediate breast reconstruction was not an option	1

**B. Reasons for implant‐based vs. autologous breast reconstruction**				
**Implant‐based** c	***N***		**Autologous** d	***N***
Autologous breast reconstruction was not an option	6		More natural outcomes	3
Shorter duration of surgery and faster recovery	3		Avoid foreign materials	3
Avoid scars and harms to other body parts	1		Opportunity to get rid of tummy	2
Fear of failure of autologous breast reconstruction	1		Complaint of implants	1
Advised by plastic surgeon	1			

*Note*: Patients could provide multiple reasons.

^a^ 11 patients.

^b^ 6 patients.

^c^ 11 patients (including 2 patients with autologous breast reconstruction who had implants before).

^d^ 6 patients.

##### 3.1.1.3 Information needed to make a decision about breast reconstruction

Patients expressed a need for objective and reliable information about BR that could be processed at their own pace and in their own time. Information should preferably be tailored to their individual situation, and preferably bundled together in one place. Patients wanted clarity about the reconstructive options that were available to them, and balanced information about the pros and cons of the options. Patients’ main questions before surgery were as follows: How will it feel and what will it look like? What will I be able to do in the period after surgery and what kind of restrictions will be imposed? When can I resume my daily activities? And, how will BR affect my daily life? Although most patients avoided emotional stories of other women, they expressed a need to learn about the experiences of other women to gain more insight into the effects of BR on their daily lives. Information about complications and less positive outcomes was also valued by patients to ensure that they have realistic expectations about BR. Although the majority of patients searched for photos to get an impression of how a reconstructed breast would look like, patients acknowledged the limited usefulness of photos in managing their expectations. Patients reported that they had underestimated the duration of the recovery period and how restricted they would be in their daily activities while recovering from surgery. Patients needed time to get used to their new bodies after surgery. They emphasized that a reconstructed breast was not simply replacing their own breast, as the appearance and sensation changed.

#### 3.1.2 Healthcare professionals

Table 4 summarizes the results among HCPs (a complete overview of the results of HCPs is provided in Supporting Information Appendix S3). The majority of HCPs (75%) were satisfied with the current information about BR provided in their hospital. All HCPs agreed that the BR decision requires active patient involvement, and considered the development of a pDA desirable (6% a little bit desirable, 52% desirable, 42% very desirable). The most frequently reported anticipated advantages of a pDA were that patients could read and process information in their own time and at their own pace, and that patients would be better informed and prepared for consultation. The most frequently reported anticipated disadvantages of the pDA were that the pDA might suggest options that are not available for an individual patient, provide patients with too much information and provide information that is not sufficiently tailored to an individual patient. Regarding the content of the pDA, the majority of HCPs preferred to include all reconstruction options available in the Netherlands, and common risk factors and complications (65%, ≥55% and ≥76%, respectively). The majority of HCPs (63%) preferred that the pDA be provided to patients during consultation with the oncological breast surgeon when the treatment options are discussed (i.e., before the first consultation with a plastic surgeon).

**Table 4:** Results of needs assessment in healthcare professionals (*N* = 33)

A. **Current information about breast reconstruction and satisfaction with information**						
Main resource for information about breast reconstruction for patients^a^						
Plastic surgeon			67%			
Internet			39%			
Oncological breast surgeon			18%			
Nurse/nurse specialist			18%			
Information leaflets			15%			
Other^b^			12%			
				**Not satisfied/disagree**	**Neutral**	**Satisfied/agree**
Satisfaction with information about breast reconstruction provided in hospital				10%	16%	74%
Patients are sufficiently informed about the possibilities of breast reconstruction				30%	15%	55%
**B. Attitudes towards shared decision making and expectations of the patient decision aid**				C. **Preferences regarding** the **content of patient decision aid**		
The decision about breast reconstruction should be made by:				Breast reconstruction options		
The patient (after seriously considering doctor's opinion)			45%	All options available in the Netherlands		65%
The patient and the doctor together			55%	Risk factors		
Doctor (after seriously considering patient's opinion)			0%	Smoking		97%
Desirability of the patient decision aid				Previous radiotherapy		97%
Very desirable			42%	Indication adjuvant radiotherapy		97%
Desirable			52%	Overweight		94%
A little bit desirable			6%	Comorbidity		94%
Not desirable			0%	Large cup size		91%
Top 3 expected advantages of a patient decision aid				Bilateral surgery		70%
Patient can process information in own time and at own pace			55%	Age (>55 years)		55%
Patient is better informed			46%	Complications		
Patient is better prepared for consultation			27%	Infections		100%
Top 3 expected disadvantages of the patient decision aid				Hematoma		100%
Might suggest options that are not available for the patient			33%	Necrosis		97%
Too much information for the patient			24%	Wound healing problems		97%
Information is not sufficiently tailored to the patient			21%	Implant‐related		97%
				Abdominal hernia/muscle weakness		76%
				Preferred timing to offer the patient decision aid		
				Consultation with the breast surgeon in which treatment options are discussed		63%

a Multiple answers allowed.

b Videos, patients, educational meetings, social worker.

### 3.2 Creation

#### 3.2.1 The target group of the patient decision aid

Based on the results of the needs assessment and discussion within the working group, we concluded that the information needs regarding BR differed between patient populations considering BR after mastectomy (i.e., patients with BC considering immediate BR, patients with BC considering delayed BR and healthy women considering BR after prophylactic mastectomy). Therefore, we focused the pDA's target group on patients with BC considering immediate BR.

#### 3.2.2 The Breast Reconstruction Patient Decision Aid

The Breast Reconstruction Patient Decision Aid (‘Borstreconstructie Keuzehulp’ in Dutch) contained three parts: a consultation sheet, an online tool and a summary sheet. The *consultation sheet* was designed for oncological breast surgeons to hand out the pDA to patients during the consultation in which the choice for BR is introduced to patients. Each sheet contained a unique login code for the online tool. The *online tool* (available at https://br.keuzehulp.nl) provided patients with an overview of reconstructive options and the pros and cons of each option, information on the effects of each option for daily life, value clarification exercises and patient stories. The online tool consisted of six modules: (1) Diagnosis. (2) Immediate breast reconstruction or not (yet)? (3) Expectations. (4) Considerations. (5) Patient Stories. (6) Summary (see Table 5 for a detailed description of each module^35^ and Supporting Information Appendix S4 for screenshots of the pDA [in Dutch]). The tool was intended for use by patients at home or at another preferred location before their consultation with a plastic surgeon. Information was presented in a way that did not favour one option over another. Patients could select the information that they want to read. The information was tailored based on the patient's treatment options (i.e., eligibility for skin and nipple‐sparing surgery, eligibility for breast‐conserving surgery and the indication for adjuvant radiotherapy). The pDA also included illustrations of different BR types. The estimated time to complete the full programme was 1 h. Upon completion of the online tool, a *summary sheet* was generated with the patient's personal considerations, preferences and questions to help inform and guide the discussion with a plastic surgeon.

**Table 5:** Overview and summary of the modules of the Breast Reconstruction Patient Decision Aid

Module	Description of module
1. Diagnosis	Based on the patient's treatment options selected on the consultation sheet by their oncological breast surgeon during the clinical encounter, patients tailor the pDA to their situation (i.e., whether or not the patient is eligible for nipple‐sparing surgery, whether or not radiotherapy is or might be necessary following surgery and whether or not the patient is eligible for breast‐conserving surgery). Based on these treatment options, specific information is shown or rephrased.
2. Immediate reconstruction or not (yet)?	Breast reconstruction options and their pros and cons are described. Options include immediate breast reconstruction, delayed breast reconstruction and no breast reconstruction.
	Information is structured as answers to the following questions: ‘What choices do I have?’, ‘What are my options?’, ‘What are the pros and cons?’, ‘How much time do I have to think?’, ‘A period without a breast?’, ‘Sparing my skin and nipple?’,^a^ ‘When can I resume my normal activities?’, ‘When is breast reconstruction finished?’, ‘What is breast‐conserving surgery?’^b^
4. Expectations	Information is provided about what patients can expect from breast reconstruction. Also, the different types of breast reconstruction and their pros and cons are described. Options include implant‐based breast reconstruction and autologous breast reconstruction.
	Information is structured as answers to the following questions: ‘What can I expect of a new breast?’, ‘What are the pros and cons of implant‐based and autologous breast reconstruction?’, ‘What if I received breast radiation in the past?’, ‘What is implant‐based breast reconstruction?’, ‘What is autologous breast reconstruction?’, ‘How will my breast look like?’, ‘How will my breast feel like?’, ‘Will this impact my body image?’, ‘What are potential complications?’, ‘What if I need breast radiation after surgery?’^c^
6. Considerations	With value clarification exercises, patients are actively encouraged to weigh the options of immediate breast reconstruction versus no immediate breast reconstruction. Furthermore, patients are invited to indicate their preference for or against immediate breast reconstruction and for the type of breast reconstruction. There is space to note questions for the plastic surgeon.
7. Patient Stories	Six short stories of patients who previously underwent mastectomy with or without breast reconstruction. The stories illustrate the experiences of these patients with decision‐making and the impact of their decision on their daily lives.
8. Summary	A summary sheet (A4 format) including the patient's personal considerations, preferences and questions for the plastic surgeon. The sheet can be saved as PDF and printed. Patients are encouraged to discuss the summary sheet with their plastic surgeon.

^a^ Information is rephrased dependent on whether or not the patient is eligible for nipple‐sparing surgery.

^b^ Section briefly describes reconstruction options after breast‐conserving surgery. Only shown if the patient is eligible for breast‐conserving surgery.

^c^ Only shown if adjuvant radiotherapy is indicated.

### 3.3 Acceptability and usability testing

Six patients, seven HCPs and seven representatives of the Dutch Breast Cancer Patient Organization participated in acceptability and usability testing. The background characteristics of the participants (*N* = 20) are provided in Table 6.

**Table 6:** Background characteristics of participants in acceptability and usability testing (*N* = 20)

			Patients (*N* = 6)	Representatives of the Dutch Breast Cancer Patient Organization (*N* = 7)
			*n*	*n*
Age (years), *M* (SD)			54.3 (13.8)	49.9 (6.1)
Level of education				
High (higher vocational/university)			5	7
Intermediate (secondary school/intermediate vocational)			1	0
Low (primary school/lower vocational)			0	0
Mastectomy			6	4
Time since mastectomy (years)				
<1			0	0
1–3			2	0
>3			4	4
Breast reconstruction				
Yes			5	3
No			1	1
Timing of breast reconstruction				
Immediate			4	2
Delayed			1	1
Type of breast reconstruction				
Implant‐based			3	1
Autologous			2	2
Combination			0	0
			**Healthcare professionals (*N*** = **7)**, ***n***	
Sex				
Female			4	
Male			3	
Profession				
Plastic surgeon			3	
Oncological breast surgeon			1	
Nurse specialist			1	
Social worker			1	
Psychologist			1	
Type of hospital				
(Breast) cancer‐specific hospital			3	
Academic medical center			3	
General hospital			1	

Abbreviations: *M*, mean; SD, standard deviation.

Patients, HCPs and representatives of the Dutch Breast Cancer Patient Organization were positive about the pDA. Participants could easily navigate through the pDA. They considered the pDA as informative and would recommend it to patients who are considering immediate BR. The patient stories were recognizable to patients, and were perceived as balanced and of added value. Participants were positive about the look and feel of the pDA. Information was perceived as well structured and understandable. While most participants appreciated the amount of information, some participants felt that it was too much. HCPs considered the pDA valuable for their patients, to prepare for consultation and to increase patient empowerment. Some HCPs expected that the pDA could also be helpful for themselves in supporting patients in decision‐making.

The most important changes made to the pDA are listed below (a detailed overview of changes is provided in Supporting Information Appendix S5):

- Text was shortened where possible;
- Information about immediate BR and its pros and cons was adjusted to more accurately reflect the situation in which a tissue‐expander is used (e.g. ‘You wake up with a reconstructed breast’ was changed to ‘You will not wake up flat’);
- The burden of recovery from autologous BR was emphasized, and information about recovery from surgery was expanded to include anticipated restrictions in daily life.

## 4 DISCUSSION

To support patients with BC in making an informed decision about immediate BR after mastectomy together with their plastic surgeon, an online patient decision aid was developed. The pDA was based on the information needs of patients and HCPs, and in accordance with international criteria for developing a high‐quality patient decision aid. The pDA was positively evaluated by patients, HCPs and representatives of the Dutch Breast Cancer Patient Organization.

Consistent with previous studies,^15^, ^16^, ^17^, ^18^, ^27^, ^43^, ^44^ the results of our needs assessment demonstrated that patients have unresolved information needs regarding their BR decision. Patients’ need for a clear overview of reconstructive options, information about the consequences of each option on patients’ daily lives and the experiences of women who previously faced the decision were consistent with information needs regarding the decision for BR described in previous studies.^17^, ^18^, ^45^ Patients’ reasons for undergoing BR, such as the desire for improved body image and appearance, and reasons against undergoing BR, such as the desire for faster recovery and avoiding increased risk of complications, were comparable to patients’ motivations for or against undergoing BR reported in previous studies.^15^, ^43^, ^45^, ^46^, ^47^, ^48^, ^49^, ^50^ Furthermore, the challenging period in which the decision about immediate BR needs to be made has been described as an obstacle for making well‐balanced decisions before.^51^

Only a limited number of studies investigated the attitudes and preferences regarding shared decision‐making in BR from the perspective of HCPs.^26^, ^27^, ^52^ The positive attitudes of HCPs towards active patient involvement and usage of the pDA were comparable to the findings of these studies.^26^, ^27^, ^52^

In developing a pDA, it is challenging to determine the appropriate amount of information. In our needs assessment, patients reported that they felt overwhelmed by the amount of information that they had to process at the time of decision‐making about BR. Therefore, we wanted to provide patients with sufficient information, without (further) overwhelming them. Individuals have different preferences in terms of the amount of information they wish to obtain when faced with a cancer‐related health threat, as some patients prefer higher levels of details than others.^53^ This emphasizes the importance of the possibility for patients to tailor the amount of information in tools like a pDA.^53^ In our pDA, patients were free to select the information they wanted to read and skip parts they did not want to read. Furthermore, we felt that we reached an appropriate amount of information in our pDA as the majority of the participants in the acceptability and usability study were satisfied with the amount of information in the pDA and members of the working group could not provide suggestions for omissions in the content of the final version of the pDA.

This study had several limitations. First, as a main limitation, selection bias may have occurred. The majority of patients and all representatives of the Dutch Breast Cancer Patient Organization who participated in the development were highly educated. Although the information in the pDA was written at a level (B1) that is understandable to most people, it remains uncertain whether the pDA is consistent with decision support needs of patients with lower educational levels, and whether the pDA is acceptable and usable for this patient group. Second, all patients participating in the acceptability and usability testing had already made their decision about BR in the past. We felt that it was inappropriate to invite recently diagnosed patients to participate in the development of the pDA and to place extra burden on them. Third, all patients participating in the acceptability and usability testing had also participated in the needs assessment.

The strength of this study was the rigorous development process, which included all relevant stakeholders from the beginning. It resulted in a pDA that incorporated information needs of both patients and HCPs and complied with international criteria for a high‐quality pDA. According to an independent group of researchers, 81% of all IPDAS criteria were fulfilled in our pDA.^54^

To investigate the pDA's impact on the decision‐making process and the decision quality, a multicentre randomized‐controlled trial is currently underway comparing use of the pDA to usual care including a widely available information leaflet.^35^, ^55^

## CONFLICT OF INTERESTS

Ir. The is cofounder and CEO of ZorgKeuzeLab. Ir. Karssen is cofounder and technical director of ZorgKeuzeLab. The remaining authors declare that there are no conflict of interests.

## AUTHOR CONTRIBUTIONS

**Jacqueline A. ter Stege**: Conceptualization, analysis (lead), investigation, methodology, project administration, visualization, writing—original draft. **Daniela Raphael**: Analysis, writing—review & editing. **Hester S. A. Oldenburg**: Conceptualization, resources, writing—review & editing. **Martine A. van Huizum**: Resources, writing—review & editing. **Frederieke H. van Duijnhoven**: Resources, writing—review & editing. **Daniela E. E. Hahn**: Resources, writing—review & editing. **Regina The**: Investigation, methodology, writing—review & editing. **Klemens Karssen**: Investigation, methodology, software, writing—review & editing. **Eveline M. L. Corten**: Resources, writing—review & editing. **Irene S. Krabbe‐Timmerman**: Resources, writing—review & editing. **Menno Huikeshoven**: Resources, writing—review & editing. **Quinten (P. Q.) Ruhé**:Resources, writing—review & editing. **Nikola (A. N.) Kimmings**: Resources, writing—review & editing. **Wies Maarse**: Resources, writing—review & editing. **Kerry A. Sherman**: Conceptualization, funding acquisition, writing—review & editing. **Arjen J. Witkamp**: Conceptualization, resources, writing—review & editing. **Leonie A. E. Woerdeman**: Conceptualization, resources, writing—review & editing. **Eveline M. A. Bleiker**: Conceptualization (lead), funding acquisition (lead), methodology (lead), project administration (lead), supervision (lead), writing—review & editing (lead).

## Supporting information

This article includes online‐only Supplemental Data.

Appendix 1. Interview script needs assessment patients.

Appendix 2. Interview script acceptability and usability testing.

Appendix 3. Results needs assessment healthcare professionals.

Appendix 4. Screenshots of pDA.

Appendix 5. Issues acceptability and usability testing and changes made.

## Data Availability

The data that support the findings of this study are available from the corresponding author upon reasonable request.
