# Supplementary material for: Development of a patient decision aid for patients with breast cancer who consider immediate breast reconstruction after mastectomy
Source: Health Expect. 2021 Oct 28;25(1):232–44. doi: 10.1111/hex.13368 (PMC8849254; doi:10.1111/hex.13368)
Supplement: Supplementary file 1 — Appendix 1. Interview script needs assessment patients. [file HEX-25-232-s005.docx]

**Appendix 1: Interviewguide Needs Assessment Patients**

**Introduction:**

- Goodmorning/good afternoon, I am [name researcher] from the Netherlands Cancer Institute. I am calling you for the interview for which we have previously been in contact. First of all, thank you for participating. This interview is about your experiences with breast reconstruction and the information provision about breast reconstruction. You already received an information letter about it. Do you have any questions about this?
- Before we start, I will first tell you something about the interview and the course of events. The interview will last approximately 45-60 minutes. I will ask you many questions about how you went through the care process and your experiences with it. There are no false answers, everything you say is valuable. To ensure that all topics are discussed, I may sometimes interrupt you. But I might also encourage you to tell more about something, if it is important to know.
- Participation is entirely voluntary and you can stop at any time. You also do not have to answer questions if you do not want to.
- Do you agree with audio recording?
- Do you have any other questions before we start?

**Interview:**

- Could you introduce yourself/could you tell something about yourself?
  - age, marital status, children, country of birth, education, occupation, hobbies, diagnosis, date of diagnosis, type of surgery and reconstruction
- Could you tell how you went through the process of breast reconstruction process? (from the beginning to the end)
- What do you remember from the conversations you had with your doctor about breast reconstruction?
  - What information did you receive?
  - What questions did you have during and after this conversation?
  - What information did you miss in the conversations about breast reconstruction?
  - What did you worry about?
- Did you feel that you had a choice about breast reconstruction?
  - If not, could you elaborate on that?
  - If so, how did you experience that choice? (Was it difficult/easy? A quick decision/did you think about it for a long time? Was there any time pressure?)
  - If so, how did your doctor involve you in this decision? How did you experience that? When?
  - Who should make the treatment decision. The doctor, you and the doctor, or the both of you?
- What were your most important considerations in making this decision?
  - What made you chose for breast reconstruction or not?
  - Immediate or delate breast reconstruction?
  - Implant-based or flap-based breast reconstruction?
- Could you also tell about your experiences with nipple-reconstruction?
  - Did you make a decision in that? How did you experience that?
- How do you look back on your decision?
  - What went different than you expected?
  - Are you satisfied with the results or did you ever experience regret?
  - Would you recommend it to other women in the same situation?
- What was the role of your partner in decision making? Others?
  - Did you discuss the decision with somebody?
  - Did that influence your decision?
- Did you look for additional information? What information were you mainly looking for?
  - contact with fellow patients, experiences of other patients, photos, chats, blogs, discussion groups, patient association, relaxation exercises
- How did you experience the period after your treatment?
  - Aftercare, recovery(period), home help, what to consider
- What would you advice other patients who just start in the trajectory of making a decision about breast reconstruction?
- We are approaching the end of the interview. Are there any important issues that haven’t been discussed so far?

**Closing**

- We have come to the end of the interview. Thank you very much for participating. How did you feel about doing it yourself? Any suggestions for us?
- When all interviews have been completed, we will write a summary report. We would like to send this to receive your feedback. Are you okay with that?
- We would like to approach you in the future for follow-up research. Are you okay with that?
- To thank you we would like to send you a book receipt. To which address can we send it?
