# Supplementary material for: Development of a patient decision aid for patients with breast cancer who consider immediate breast reconstruction after mastectomy
Source: Health Expect. 2021 Oct 28;25(1):232–44. doi: 10.1111/hex.13368 (PMC8849254; doi:10.1111/hex.13368)
Supplement: Supplementary file 2 — Appendix 2. Interview script acceptability and usability testing. [file HEX-25-232-s001.docx]

**Appendix 2: Interview script acceptability and usability testing**

| Background (Date, age, profession) |  |
| --- | --- |
| What did you expect of the decision aid (before usage)? |  |
| What was your first impression of the decision aid? |  |
| What do you consider as positive? |  |
| What could be improved? |  |
| What do you think of the patient stories ? |  |
| What do you think about the amount of information? What information can be omitted? What information did you miss? |  |
| *Patients + Representatives:*  Would you recommend the decision aid to women with breast cancer deciding about breast reconstruction?  *Healthcare professionals:*  Would you offer this to your patients?   - What barriers do you expect? - What would facilitate using the decision aid? |  |
| Do you have any other remarks or considerations that you would like to share? |  |
