# Supplementary material for: Development of a patient decision aid for patients with breast cancer who consider immediate breast reconstruction after mastectomy
Source: Health Expect. 2021 Oct 28;25(1):232–44. doi: 10.1111/hex.13368 (PMC8849254; doi:10.1111/hex.13368)
Supplement: Supplementary file 3 — Appendix 3. Results needs assessment healthcare professionals. [file HEX-25-232-s004.docx]

**Appendix 3: Results of needs assessment in healthcare professionals (N=33)**

**A ) Information provision about breast reconstruction**

Main information resource about breast reconstruction for patients according to healthcare professionals (N=33)

Note. X-axis= Number of times that information resource was reported. Multiple answers were allowed.

|  | **No.** | **%** |
| --- | --- | --- |
| **How are patients informed about breast reconstruction in your hospital?** |  |  |
| oral information by plastic surgeon | 32 | 100 |
| oral information by oncological surgeon | 25 | 78 |
| information leaflet of hospital | 24 | 75 |
| oral information by breast cancer nurse | 23 | 72 |
| information leaflet of Dutch Cancer Society | 17 | 53 |
| oral information by nurse specialist | 13 | 41 |
| website of hospital | 11 | 33 |
| website Dutch Breast Cancer Patient Organization | 7 | 22 |
| website of Dutch Society for Plastic Surgeons | 6 | 19 |
| Others | 6 | 19 |
| B-Bewust website | 5 | 16 |
| website keuzehulp.info | 2 | 6 |
| website kanker.nl | 1 | 3 |
| information center in hospital | 1 | 3 |
| website of other hospital | 1 | 3 |
| **Average number of consultations with plastic surgeon for decision-making about (immediate) breast reconstruction following breast cancer diagnosis** |  |  |
| one consultation | 9 | 28 |
| two consultations | 17 | 53 |
| three or more consultations | 1 | 3 |
| I don't know | 5 | 16 |

N=32 as questions were inappropriate for 1 healthcare professional who did not work in a hospital

**B ) Satisfaction with current information about BR according to healthcare professionals**

|  |  |  | **No. (%)** | | | | |
| --- | --- | --- | --- | --- | --- | --- | --- |
|  | **N** | **M (SD)** | **1** | **2** | **3** | **4** | **5** |
| Satisfaction with information about breast reconstruction | 32 | 3.3 (0.8) | 0 (0) | 7 (22) | 11 (34) | 13 (41) | 1 (3) |
| Satisfaction with information about breast reconstruction provided in your hospital* | 31* | 3.7 (0.8) | 0 (0) | 3 (10) | 5 (16) | 20 (65) | 3 (10) |
| Patients are sufficiently informed about the possibilities of breast reconstruction | 33 | 3.4 (1.1) | 0 (0) | 10 (30) | 5 (15) | 13 (39) | 5 (15) |
| The information about breast reconstruction is reliable^1^ | 33 | 3.4 (0.9) | 0 (0) | 5 (15) | 13 (39) | 12 (36) | 3 (9) |

1 = ‘not satisfied at all’ or ‘completely disagree’, 3 = ‘neutral’, 5 = ‘very satisfied’ or ‘completely agree’

*2 missing; 1 not applicable, 1 missing

^1^Due to a data storage issue, the number of healthcare professionals in categories 2 (disagree) and 4 (agree) might have been 6 and 11, respectively, resulting in a mean score of 3.5.

**C) Attitudes of healthcare professionals (N=33) on patient involvement in decision-making about breast reconstruction**

|  |  |  | **No. (%)** | | | | |
| --- | --- | --- | --- | --- | --- | --- | --- |
|  | **N** | **M (SD)** | **1** | **2** | **3** | **4** | **5** |
| Better informed patients facilitate the decision-making process^1^ | 33 | 4.3 (0.7) | 0 (0) | 1 (3) | 2 (6) | 15 (45) | 15 (45) |
| Better-informed patients complicate the decision-making process^2^ | 33 | 2.2 (1.2) | 13 (39) | 9 (27) | 6 (18) | 3 (9) | 2 (6) |
| If a patient does not want to be involved in decision-making, the doctor must still try to involve the patient | 33 | 4.0 (0.7) | 0 (0) | 1 (3) | 5 (15) | 19 (58) | 8 (24) |
| Every patient must be informed about the reconstructive options that apply to her, also if she has to be referred to another hospital for this option | 33 | 4.9 (0.4) | 0 (0) | 0 (0) | 0 (0) | 5 (15) | 28 (85) |

1 = ‘completely disagree’, 3 = ‘neutral’, 5 = ‘completely agree’

^1^Due to a data storage issue, the number of healthcare professionals in categories 2 (disagree) and 4 (agree) might have been 0 and 16, respectively, resulting in a mean score of 4.4.

^2^Due to a data storage issue, the number of healthcare professionals in categories 2 (disagree) and 4 (agree) might have been 10 and 2, respectively, resulting in a mean score of 2.1.

|  |  | **N** | **%** |
| --- | --- | --- | --- |
| **The decision about breast reconstruction should be made by..** | |  |  |
| patient |  | 2 | 6 |
| patient after seriously considering the doctor's opinion | | 13 | 39 |
| patient and doctor together | | 18 | 55 |
| doctor after seriously considering the patient's opinion | | 0 | 0 |
| doctor |  | 0 | 0 |

**Facilitators and barriers for patient involvement in decision-making about breast reconstruction according to healthcare professionals (N=33)**

|  | **Facilitators** | **Barriers** |
| --- | --- | --- |
| **Patient-related** | Well-informed (5) | Insufficiently or erroneously informed (4) |
|  | Active/asks questions (2) | Emotional state (3) |
|  | Aware of preference-sensitive nature of decision (1) | Limited understanding and/or language barrier (3) |
|  | Trust in doctor (1) | No headspace to think about breast reconstruction (2) |
|  | High educational level (1) | Demanding (1) |
|  | Feeling of social support (1) | Subassertive (1) |
|  |  | Unrealistic expectations (1) |
|  |  | Difficulties to foresee consequences (1) |
| **Physician-related** | Informs about options and pros and cons (3) | Provides personal opinion (instead of options) (1) |
|  | knowledge of and attitude towards breast reconstruction of oncological surgeon (3) |  |
|  | emphasizes personal nature of decision / importance shared decision making (2) |  |
|  | Is involved (1) |  |
| **Organization of care** | Easy access to (supportive) care (3) | Limited time to decide / for consultation (2) |
|  | More than one consultation (2) |  |
|  | Time to think after consultation (1) |  |
| **Information** | Provision of visual materials (2) | Large amount of information (1) |
|  | Experiences of other patients (1) |  |
| **Relatives** | Presence/involvement of relatives in consultation (2) | Dominant partner (2) |
| **Decision** |  | Large number of options (2) |

**D) Attitudes of healthcare professionals (N=33) towards development of breast reconstruction decision aid**

|  | **N** | **%** |
| --- | --- | --- |
| **Desirability of the development of decision aid** |  |  |
| not at all desirable | 0 | 0 |
| not desirable | 0 | 0 |
| a little bit desirable | 2 | 6 |
| desirable | 17 | 52 |
| very desirable | 14 | 42 |

**Expected advantages and disadvantages of breast reconstruction decision aid according to healthcare professionals (N=33)**

| **Expected advantages** | **Expected disadvantages** |
| --- | --- |
| Patient can process information in own time and at own pace (18) | Might suggest options that are not available for patient (11) |
| Patient is better informed (15) | Too much information for patient (8) |
| Patient is better prepared for consultation (9) | Information is not sufficiently tailored to patient (7) |
| Provides objective information (5) | Not accessible for all patients (6) |
| Higher patient satisfaction and less regret (4) | Patients might feel conflicted about decision or ‘left alone’ to make the decision (5) |
|  | No possibility to provide immediate feedback (4) |
| Enables well-informed decision (4) | Patient might rely too much on outcome of decision aid (3) |
| Supports doctor in discussing pros and cons and clarifying patients' values (3) | Too little attention for emotional aspects in decision-making (3) |
| Provides standardized information (3) | Some patients do not want to make / be involved in the decision (2) |
| Saves time during consultation (3) | Increases consultation time (2) |
|  | Might confuse patients (2) |
| Provides reliable information (2) | Leads to unrealistic expectations (2) |
| Provides tailored information (2) | No substitute for consultation (2) |
| Patient has realistic expectations (2) | Extra burden for patient (2) |
| Fastens decision-making process (2) | Information must constantly be kept up-to-date (2) |
| Increases patient involvement (2) | Partner might be involved to little (1) |
| Increases choice awareness (1) | Might provide subjective information (1) |
| Reduces stress (1) | Negative impact on patient communication if plastic surgeon is not aware of or does not support content of decision aid (1) |
|  |  |
| Pictures and examples (1) |  |

**E) Preferences for content and timing of the patient decision aid according to healthcare professionals (N=33)**

|  |  | **N** | **%** |
| --- | --- | --- | --- |
| **What should be discussed in the patient decision aid?** | |  |  |
| ***Breast reconstruction options*** | |  |  |
| all breast reconstructive options offered worldwide | | 5 | 15 |
| all breast reconstructive options offered in The Netherlands | | 20 | 61 |
| all breast reconstructive options discussed in guideline | | 5 | 15 |
| all breast reconstructive options offered in hospital | | 0 | 0 |
| other | | 3 | 9 |
| ***Risk factors*** | |  |  |
| smoking | | 32 | 97 |
| previous radiotherapy | | 32 | 97 |
| indication adjuvant radiotherapy | | 32 | 97 |
| overweight | | 31 | 94 |
| comorbidity (e.g. diabetes, high blood pressure) | | 31 | 94 |
| large cup size | | 30 | 91 |
| bilateral surgery | | 23 | 70 |
| age (>55 years) | | 18 | 55 |
| other* | | 5 | - |
| ***Complications*** | |  |  |
| infections | | 33 | 100 |
| hematoma | | 33 | 100 |
| necrosis | | 32 | 97 |
| wound healing problems | | 32 | 97 |
| implant-related (including capsular contracture) | | 32 | 97 |
| abdominal hernia/muscle weakness (DIEP-flap) | | 25 | 76 |
| other** | | 28 | - |
| **Preference regarding visual material in patient decision aid** | |  |  |
| no photos or illustrations | | 2 | 6 |
| photos |  | 0 | 0 |
| illustrations | | 7 | 21 |
| photos and illustrations | | 24 | 73 |
| **Preferred timing to offer patient decision aid to patient** | |  |  |
| consultation in which diagnosis is communicated | | 4 | 13 |
| consultation with oncological breast surgeon in which treatment options are discussed | | 20 | 63 |
| consultation with plastic surgeon | | 4 | 13 |
| other*** | | 4 | 13 |
| missing | | 1 | 3 |

*Other risk factors (all reported once): quality of skin and tissue and scars, surgeries of abdomen or back in the past, surgeries of breast in the past, mental state, history of thromboembolism.

**Other complications (number of times reported): failure (7), reoperations (3), pulmonary embolism (3), spasm of pectoralis major (2), asymmetry (2), abnormal scarring (2), regret ( 1), thrombosis leg (1), pain (1), malpositioning of prosthesis (1), prosthetic rupture (1), seroma (1), dogears (1), impact of complications on adjuvant treatment (1), functional problem (1). ***other = prior to consultation with plastic surgeon

**F ) Healthcare professional’s (N=33) beliefs about patients' most important motivations to decide for a specific breast reconstruction option**

|  | | **No.** |
| --- | --- | --- |
| **Breast reconstruction (versus no breast reconstruction)** | |  |
|  | retain/restore femininity | 12 |
|  | avoid to be 'flat' | 8 |
|  | retain/restore identity and self-image | 4 |
|  | more clothing possibilities | 4 |
|  | cosmetics | 3 |
|  | symmetry | 3 |
|  | self-confidence | 2 |
|  | avoid use of external breast prosthesis | 2 |
|  | keep/restore breast shape | 2 |
|  | feel 'whole' | 2 |
|  | repair of mutilation | 2 |
|  | keep cleavage | 1 |
|  | attractiveness | 1 |
|  | prevent stigma | 1 |
| **No breast reconstruction** | |  |
|  | too much hassle | 8 |
|  | avoid additional operations | 7 |
|  | avoid additional risks for complications | 6 |
|  | avoid foreign materials and/or silicones | 4 |
|  | no need for breast reconstruction / reconstruction is considered unnecessary | 3 |
|  | breasts do not determine patients’ femininity or well-being | 2 |
|  | indication for adjuvant radiotherapy | 2 |
|  | faster recovery | 2 |
|  | age | 2 |
|  | recover from breast cancer first | 2 |
|  | avoid additional scars | 2 |
|  | experiences from relatives | 1 |
|  | insufficient information about possibilities | 1 |
|  | too much information | 1 |
|  | accepts impact of disease | 1 |
|  | considers 'flat' as beautiful | 1 |
|  | stress overload | 1 |
| **Immediate breast reconstruction (versus delayed breast reconstruction)** | | |
|  | fewer surgeries | 16 |
|  | avoid being 'flat' after surgery | 10 |
|  | sparing breast skin | 3 |
|  | superior cosmetic result | 2 |
|  | medically superior | 1 |
|  | feeling less mutilated | 1 |
|  | superior for psychological functioning | 1 |
|  | less confrontation with loss of breast | 1 |
|  | prevent stigma | 1 |
|  | feeling feminine immediately after surgery | 1 |
|  | return to normal as fast as possible | 1 |
| **Delayed breast reconstruction (versus immediate breast reconstruction)** | | |
|  | complete oncological treatment first | 12 |
|  | no headspace to think about breast reconstruction after diagnosis | 8 |
|  | need more time to decide | 2 |
|  | wait to see how life is experienced without breast | 2 |
|  | adjuvant radiotherapy | 2 |
|  | fear for adjuvant therapy | 1 |
|  | fear for silicone implants | 1 |
|  | way to reach preferred results | 1 |
|  | doctor's advice | 1 |
|  | letting the wound heal first | 1 |
|  | avoid additional risks and complications | 1 |
|  | belief of superior cosmetic results | 1 |
|  | presence of surgical risk factors | 1 |
| **Implant-based breast reconstruction (versus autologous breast reconstruction)** | | |
|  | relative simple operation | 18 |
|  | no scarring of other body parts | 13 |
|  | shorter surgery | 9 |
|  | faster recovery | 5 |
|  | not eligible for autologous breast reconstruction | 4 |
|  | opportunity to increase cup size | 3 |
|  | keep the possibility of autologous breast reconstruction in case of complications | 1 |
|  | can be performed in all hospitals | 1 |
|  | most patients are satisfied with implant-based breast reconstruction | 1 |
|  | If this option is better for medical reasons | 1 |
| **Autologous breast reconstruction (versus implant-based breast reconstruction)** | | |
|  | more natural outcomes (e.g. temperature, look, feel, aging of breast) | 19 |
|  | avoid foreign materials/silicones in body | 19 |
|  | no more surgeries required once completed/sustainability | 6 |
|  | superior cosmetic results | 6 |
|  | opportunity to get rid of redundant body tissue | 3 |

Note. Multiple answers were allowed
