# Supplementary material for: Development of a patient decision aid for patients with breast cancer who consider immediate breast reconstruction after mastectomy
Source: Health Expect. 2021 Oct 28;25(1):232–44. doi: 10.1111/hex.13368 (PMC8849254; doi:10.1111/hex.13368)
Supplement: Supplementary file 4 — Appendix 4. Screenshots of pDA. [file HEX-25-232-s003.docx]

**Appendix 4: Screenshots of the Breast Reconstruction Patient Decision Aid**
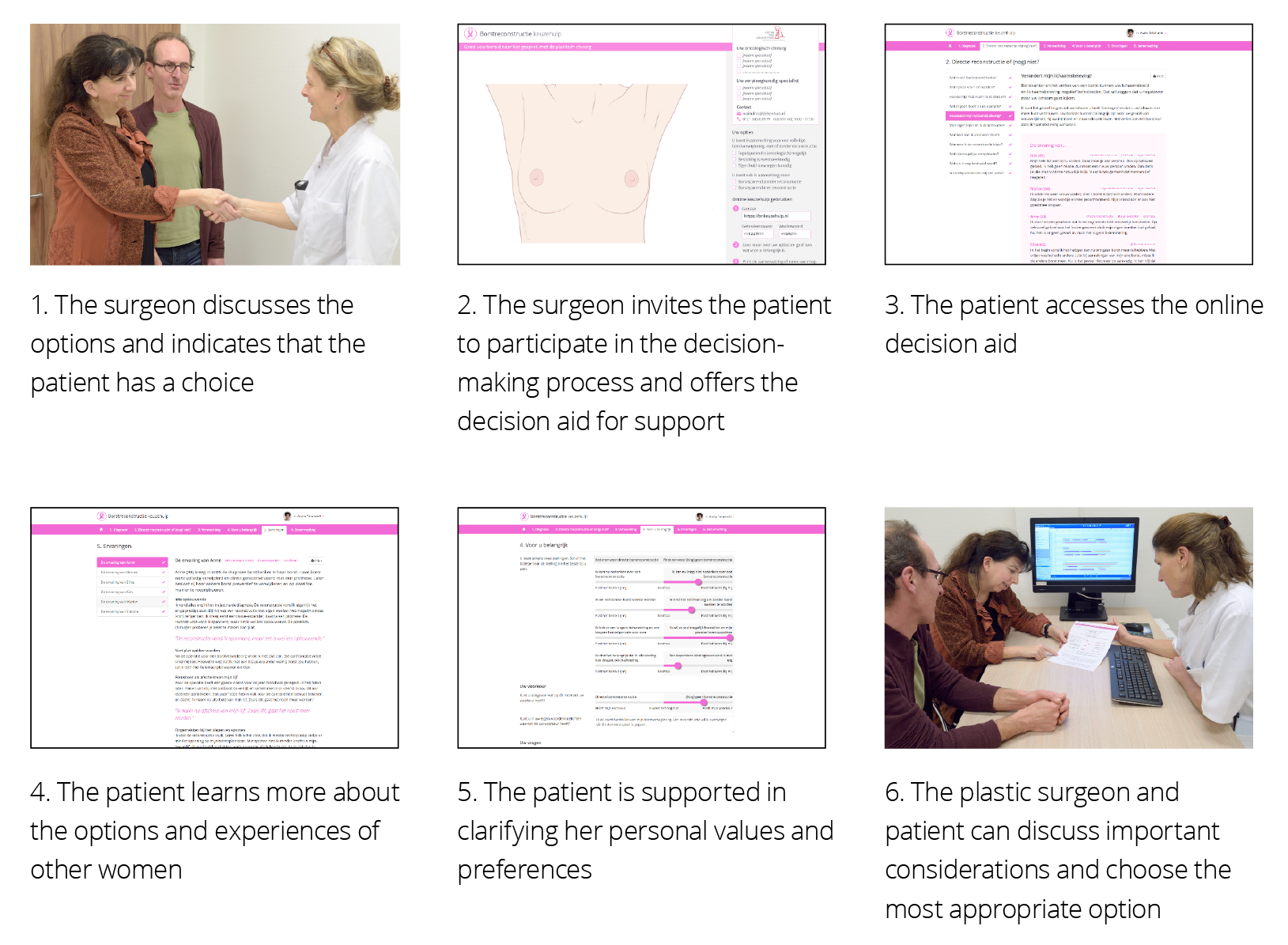


Fig 1. Roadmap illustrating how the breast reconstruction decision aid is used


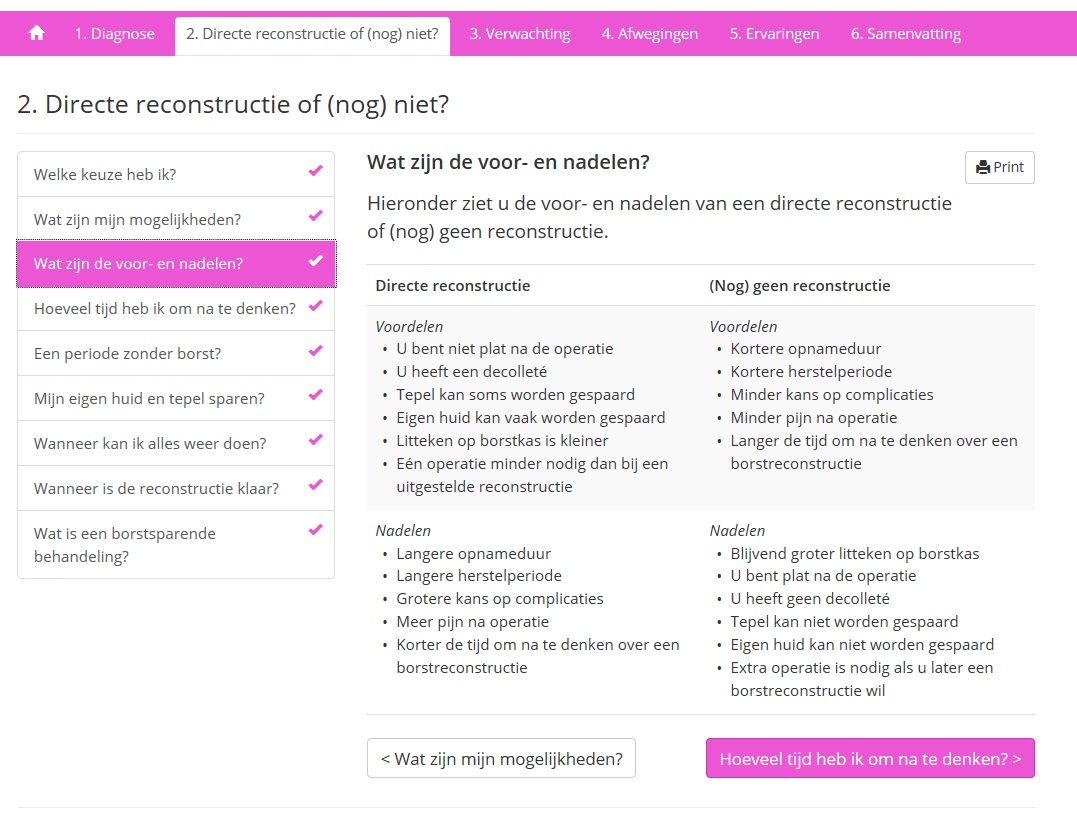


Fig 2. Module 2: Immediate reconstruction or not (yet)?, table with pros and cons of options (in Dutch)


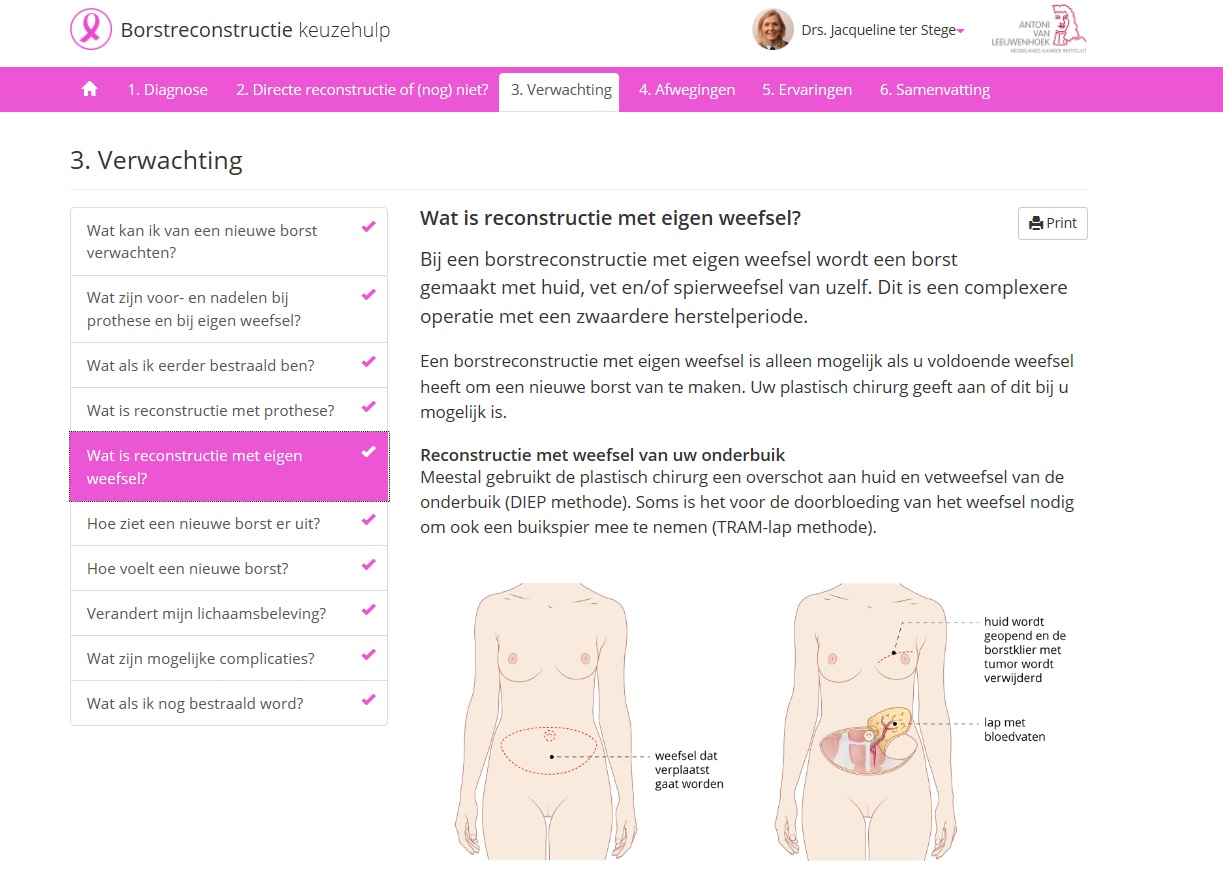


Fig 3. Module 3: Expectations, ‘What is autologous breast reconstruction?’ (in Dutch)


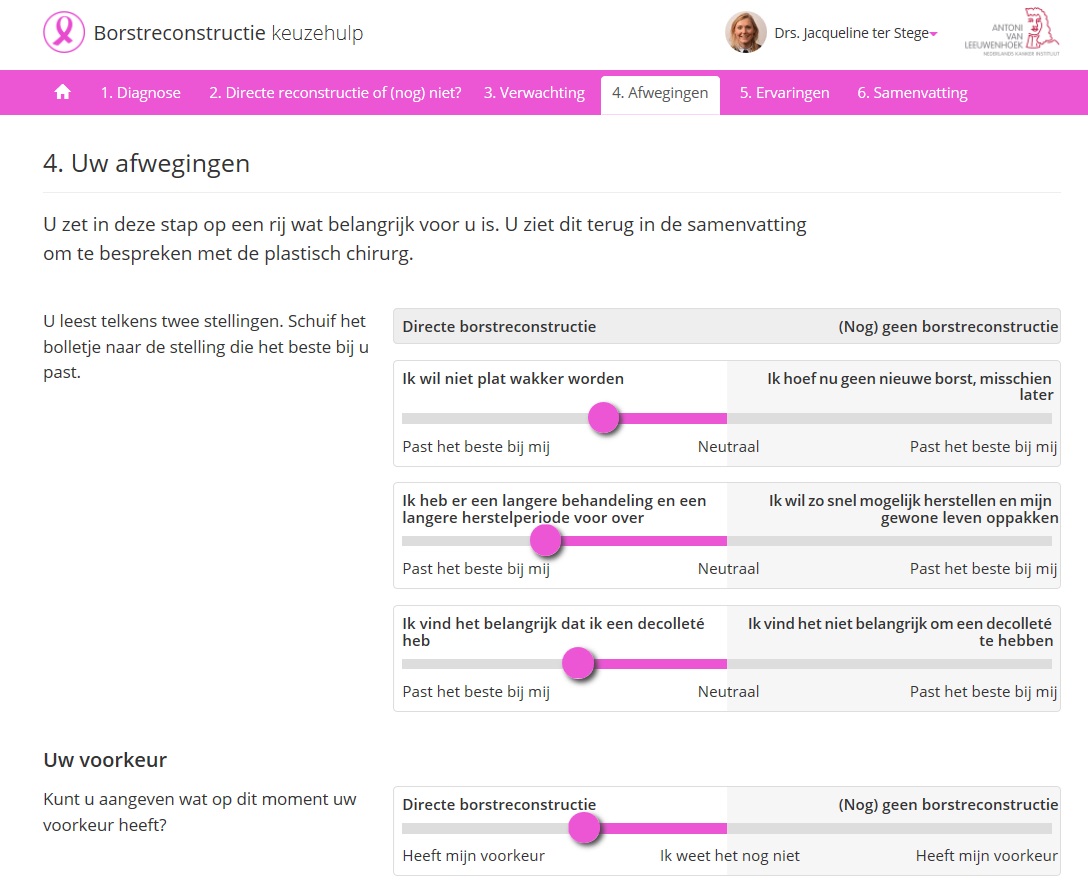


Fig 4. Module 4: Considerations, including value clarification exercises (in Dutch)


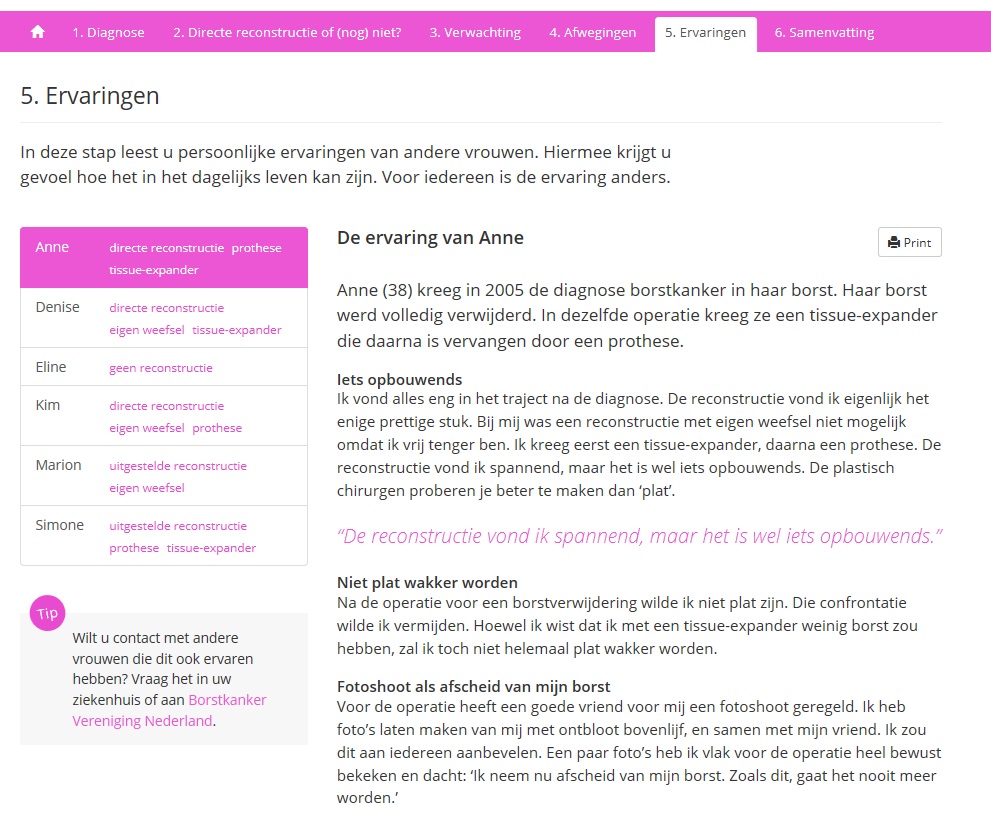


Fig 5. Module 5: Patient Stories (in Dutch)


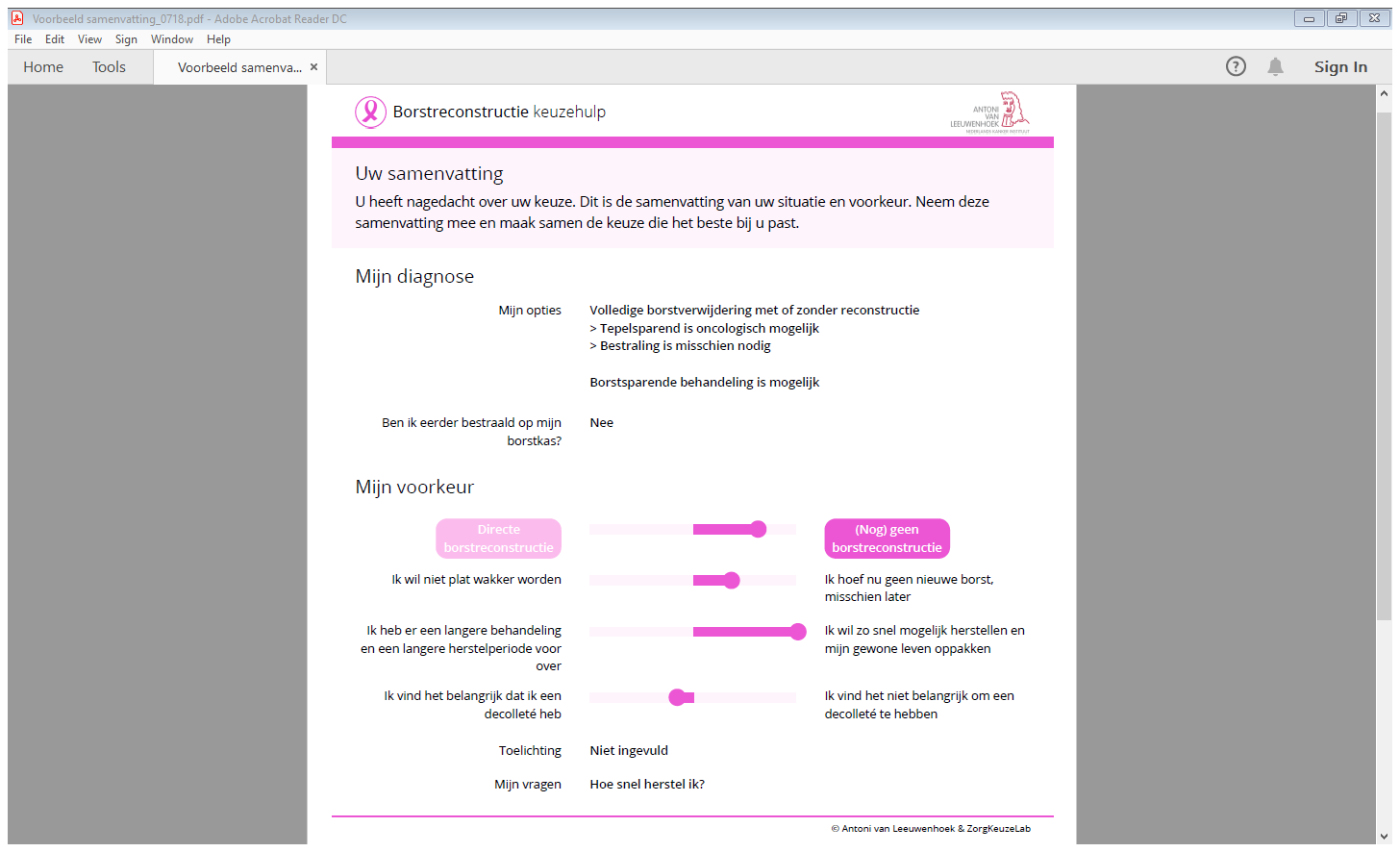


Fig 6. Summary sheet (generated in Module 6) including patient’s personal considerations, preferences and questions
