# Supplementary material for: Development of a patient decision aid for patients with breast cancer who consider immediate breast reconstruction after mastectomy
Source: Health Expect. 2021 Oct 28;25(1):232–44. doi: 10.1111/hex.13368 (PMC8849254; doi:10.1111/hex.13368)
Supplement: Supplementary file 5 — Appendix 5. Issues acceptability and usability testing and changes made. [file HEX-25-232-s002.docx]

**Appendix 5: Overview of issues raised in acceptability and usability testing and changes made to the patient decision aid**

| **No.** | **Module** | **FAQ** | **Topic** | **Issue** | **Change** |
| --- | --- | --- | --- | --- | --- |
| 1 | NA | NA | Language/comprehension | The term oncological may be difficult to understand for patients | The word oncological was avoided |
| 2 | NA | NA | Language/comprehension | The amount of information was considered as too large by some | Texts were restructured with subheadings, divided into separate sections, and shortened where possible |
| 3 | 1 | NA | Content | The treatment option 'breast conserving surgery with or without BR is possible' is confusing, as BR refers to BR after mastectomy throughout the tool | Changed to 'Breast conserving surgery is possible' |
| 4 | 1 | NA | Content | The question to fill in particularities that should be taken into account is difficult to complete at this point | Question was moved to module 4 |
| 5 | 2 | What choices do I have? | Content | An exemplar reason to have immediate BR (i.e. I want to wake up with a reconstructed breast) does not hold for patients with BR with TE | Exemplar reason for immediate BR changed to: I don't want to wake up flat |
| 6 | 2 | What are my options? | Content | The information about immediate BR does not accurately correspond to the situation in which a TE is placed prior to the final implant | The option of immediate BR with a TE is introduced earlier in the pDA, and Information was adjusted to better reflect this situation |
| 7 | 2 | What are the pros and cons? | Content | It is unclear how much longer the hospital stay is in case of an immediate BR | No change made because of variability between hospitals |
| 8 | 2 | What are the pros and cons? | Content | The pro of immediate BR ‘wider choice of clothing’ can be perceived normative | This pro was removed |
| 9 | 2 | How much time do I have to think? | Content | Suggestion to add the advice to take sufficient time for decision-making | Advice was added |
| 10 | 2 | How much time do I have to think? | Content | Suggestion to add that patients need to wait at least 6 months after mastectomy for delayed BR | Not added as working group disagreed |
| 11 | 2 | A period without a breast? | Content | Suggestion to add the advice to ask for special information days about external prosthesis in hospital | Not added to not further increase the amount of information in this section |
| 12 | 2 | A period without a breast? | Content | Suggestion to add common inconveniences of wearing an external prosthesis | Common inconveniences were added (i.e. It may feel heavy, fall out of the bra, sometimes still be visible and you may sweat more) |
| 13 | 2 | Sparing my skin and nipple? | Content | Information is provided about lost/reduced sensation of the beast and breast skin in case of immediate BR. However, it is unclear what happens in case of mastectomy without immediate BR | Text was changed to clarify (“After mastectomy, sensation is lost or reduced. Also after breast reconstruction, you will not or barely have sensation in your skin if it is touched") |
| 14 | 2 | Sparing my skin and nipple? | Content | Suggestion to add that scars are red and big shortly after surgery, but that this disappears after a while | No information added |

**Appendix 5: Overview of issues raised in acceptability and usability testing and changes made to the patient decision aid (continued)**

| **No.** | **Module** | **FAQ** | **Topic** | **Issue** | **Change** |
| --- | --- | --- | --- | --- | --- |
| 15 | 2 | When can I resume my normal activities? | Content | Information does not reflect the true burden of the recovery period after surgery | We added: You may need help at home with getting dressed and with your household. |
| 16 | 2 | When can I resume my normal activities? | Language/comprehension | A fragment of patient story dealing about a scar on patient's belly is unclear, as A-BR has not yet been explained | The fragment was left out from patient story at that section |
| 17 | 2 | When can I resume my normal activities? | Content | Duration of complaints following surgery in patient story (mastectomy without BR) was not considered representative | The duration of the period was left out from patient story |
| 18 | 2 | When is breast reconstruction finished | Content | Suggestion to specify possible adjustments to the breast in the phrase 'sometimes the healthy breast is adjusted' (i.e. reduced, lifted, etcetera) | No change made |
| 19 | 3 | ‘What can I expect of a new breast? | Content | Suggestion to leave out the information that A-BR can also be performed after I-BR, because of doubts whether this is covered by health insurance | No change made |
| 20 | 3 | What are the pros and cons of implant-based and autologous BR? | Content | Suggestion to add ‘does not require replacements in the future’ as pro for A-BR | Pro was added. ‘Real chance for needing a replacement in the future’ was added as a con for I-BR |
| 21 | 3 | What are the pros and cons of implant-based and autologous BR? | Content | Information about differences in the recovery period between A-BR and I-BR is lacking | Information was added, emphasizing the burden of recovery after A-BR |
| 22 | 3 | What are the pros and cons of implant-based and autologous BR? | Content | Suggestion to specify duration of surgery and recovery period | Not specified because of variability |
| 23 | 3 | What are the pros and cons of implant-based and autologous BR? | Content | Suggestion to add con of immediate I-BR: Prosthesis can move if not preceded by a TE | Information was not added as it is rare and we refer to an additional information resource containing this information |
| 24 | 3 | What are the pros and cons of implant-based and autologous BR? | Content | Suggestion to add ‘tummy tuck’ as pro of A-BR | Not added, as this was not considered most important pro of A-BR, only applicable for specific type of A-BR, and this aspect is highlighted in a patient story |
| 25 | 3 | What are the pros and cons of implant-based and autologous BR? | Content | Change con of A-BR to 'Can’t be performed in every hospital’ instead of ‘Complex surgery’ | Not changed |
| 26 | 3 | What are the pros and cons of implant-based and autologous BR? | Content | Suggestion to add that immediate BR is not offered in every hospital and that you might have to change from hospital. | Not changed |
| 27 | 3 | What is implant-based breast reconstruction? | Content | Specify the impact of having a TE on traveling | ‘This means that you will have to come to the hospital multiple times’ was added |

**Appendix 5: Overview of issues raised in acceptability and usability testing and changes made to the patient decision aid (continued)**

| **No.** | **Module** | **FAQ** | **Topic** | **Issue** | **Change** |
| --- | --- | --- | --- | --- | --- |
| 28 | 3 | What is implant-based breast reconstruction? | Content | Suggestion to add that filling a TE might cause pain | Not added (to be as concise as possible, and was not perceived of high importance for the decision for a TE) |
| 29 | 3 | What is implant-based breast reconstruction? | Lay-out | Reference to surgical package insert seemed to be in the wrong place | Reference was replaced |
| 30 | 3 | What is autologous breast reconstruction? | Content | Suggestion to place greater emphasis on the burden of recovery after A-BR | Burden of recovery period was emphasized, and patient story illustrating recovery period was added |
| 31 | 3 | Will this impact my body image?’ | Content | Text was phrased in a negative way | Text was rephrased in a more positive way |
| 32 | 3 | Will this impact my body image?’ | Content | The text about impact of BC diagnosis 'you may feel that your body has failed you and that you can no longer trust your body' was considered irrelevant for the decision about BR | Information was left out |
| 33 | 3 | What are potential complications? | Content | Pulmonary embolism is missed as complication | Not added, as this is very rare and a complication of surgery in general |
| 34 | 3 | What are potential complications? | Content | Information raises questions about the probabilities of complications | We added the suggestion to ask your plastic surgeon for individual risk estimates. Probabilities of complications were not added, because there was no consensus on the numbers (i.e. risk for complications differs per patient, very broad range, and differences per hospital) |
| 35 | 3 | What if I need breast radiation following surgery? | Content | Suggestion to add information about the risks of radiotherapy after immediate I-BR | Text was rewritten to: Radiation treatment after reconstruction is more likely to cause scarring. As a result, there is a good chance that after a few years you will need surgery in your reconstructed breast |
| 36 | 3 | What if I need breast radiation following surgery? | Content | Text ‘If it is clear before surgery that you will need radiotherapy after surgery, a delayed breast reconstruction is often advised' is not in line with current practice | Text changed to: 'If it is clear that you will need radiotherapy after surgery, delayed breast reconstruction can be advised' |
| 37 | 4 | Considerations | Content | Value statements "I can think about breast reconstruction at this time" vs. "I can't think about it yet" (leaning towards immediate BR vs. not, respectively) can be perceived normative, and are not correct | Statements were left out |
| 38 | 4 | Considerations | Content | Value statement "I don't mind waking up without a breast" (leaning towards not having immediate BR) was considered inappropriate | Statement was changed to "I don't need a new breast now, maybe later" |

**Appendix 5: Overview of issues raised in acceptability and usability testing and changes made to the patient decision aid (continued)**

| **No.** | **Module** | **FAQ** | **Topic** | **Issue** | **Change** |
| --- | --- | --- | --- | --- | --- |
| 39 | 4 | Considerations | Content | Value statement 'It is important to me, that I am able to wear all types of clothing, including swimwear' (leaning towards immediate BR) was considered normative | Value statement was changed into "It is important for me to have a cleavage" |
| 40 | 4 | Considerations | Content | Suggestion to refer to existing resource (Question Prompt Lists) of Dutch breast cancer patient organization | Linkage was added |
| 41 | 5 | NA | Content | There is no story of a patient with complications among the patient stories | Listed as a wish for further development of pDA |
| 42 | 5 | NA | Content | Suggestion to add reference to peer contact team of Dutch breast cancer patient organization | Reference was added |

Abbreviations. A-BR = Autologous breast reconstruction. BR = Breast reconstruction. I-BR = Implant-based breast reconstruction. pDA = patient decision aid. TE = Tissue-expander.
